# Addition of ROCK Inhibitors Alleviates Prostaglandin-Induced Inhibition of Adipogenesis in 3T3L-1 Spheroids

**DOI:** 10.3390/bioengineering9110702

**Published:** 2022-11-17

**Authors:** Yosuke Ida, Tatsuya Sato, Araya Umetsu, Megumi Watanabe, Masato Furuhashi, Fumihito Hikage, Hiroshi Ohguro

**Affiliations:** 1 Departments of Ophthalmology, Sapporo Medical University School of Medicine, Sapporo 060-8556, Japan; 2 Departments of Renal and Metabolic Medicine, Sapporo Medical University School of Medicine, Sapporo 060-8556, Japan; 3 Departments of Cellular Physiology and Signal Transduction, Sapporo Medical University School of Medicine, Sapporo 060-8556, Japan

**Keywords:** 3T3-L1 cell, upper eyelid sulcus (DUES) deepening, 3-dimensional (3D) tissue culture, Rho-kinase, ROCK, ROCK inhibitor

## Abstract

To elucidate the additive effects of the ROCK inhibitors (ROCK-i), ripasudil (Rip) and Y27632 on bimatoprost acid (BIM-A), a prostaglandin analog (PG), on adipose tissue, two- and three-dimensional (2D or 3D) cultures of 3T3-L1 cells, the most well characterized cells in the field of lipid research, were used. The cells were subjected to a variety of analyses including lipid staining, real-time cellular metabolic analysis, the mRNA expressions of genes related to adipogenesis and extracellular matrices (ECMs) as well as the sizes and physical properties of the 3D spheroids by a micro-squeezer. BIM-A induced strong inhibitory effects on most of the adipogenesis-related changes in the 2D and 3D cultured 3T3-L1 cells, including (1) the enlargement and softening of the 3D spheroids, (2) a dramatic enhancement in lipid staining and the expression of adipogenesis-related genes, and (3) a decrease in mitochondrial and glycolytic metabolic function. By adding ROCK-i to the BIM-A, most of these BIM-A-induced effects were cancelled. The collective findings reported herein suggest that ROCK-i eliminated the PG-induced suppression of adipogenesis in the 3T3-L1 cells, accompanied by the formation of enlarged 3D spheroids. Such effects of adding ROCK-i to a PG in preadipocytes on cellular properties appear to be associated with the suppression of PG-induced adverse effects, and provide additional insight into our understanding of lipid-related research.

## 1. Introduction

It is well known that adipocytes are involved as master regulators of energy homeostasis by storing large amounts of triacylglycerols or by mobilizing lipids in the cases of an excess of energy or nutritional deficiencies, respectively [1,2]. Adipocyte differentiation, a process in which preadipocytes proliferate and then differentiate into mature adipocytes, is precisely coordinated by various physiological mechanisms, including the gene expression of several adipogenesis-related factors and hormone sensitivity. It is also regulated by several intracellular signaling pathways [3]. Among these factors, prostaglandin derivatives (PGs) secreted from adipocytes both positively and negatively regulate adipogenesis [4]. For example, PGI2 promotes the progression of the differentiation of adipocytes into preadipocytes through PGI2 receptors (IP) by enhancing the expression of important transcription factors that are involved in the activation of the early stage of adipogenesis. These include the CCAAT/enhancer binding protein (C/EBP) β and δ [5,6], resulting in the subsequent activation of the expression of peroxisome proliferator-activated receptor γ (*PPARγ*), a critical transcription factor that promotes the maturation of adipocytes via adipogenesis. In fact, it was revealed that PGI2 stimulates high-fat diet (HFD)-induced obesity through IP receptors in adipocytes [7]. It is also known that PGD2 activates adipogenesis through a chemoattractant receptor-homologous molecule that is expressed on type 2 T helper (Th2) cells (CRTH2/DP2 receptors) and *PPARγ* [8,9,10,11]. Meanwhile, in contrast to PGI2 and PGD2, PGF2α and PGE2 inhibit the early stage of adipogenesis by increasing the COX-2-mediated production of PGF2α and PGE2 through their respective receptors [4].

Alternatively, it is also well known that Rho kinases (ROCKs), major downstream effectors of small GTPase RhoA [12,13,14,15], represent another pivotal regulatory factor modulating adipogenesis. In fact, the ROCK family, including ROCK1 and ROCK2, has recently attracted interest as a potential therapeutic target for the treatment of metabolic disorders, based upon the fact that upregulated ROCK activity is involved in the pathogenesis of nearly all of the metabolic syndromes including obesity, insulin resistance, dyslipidemia and hypertension [16].

**Figure 1:**
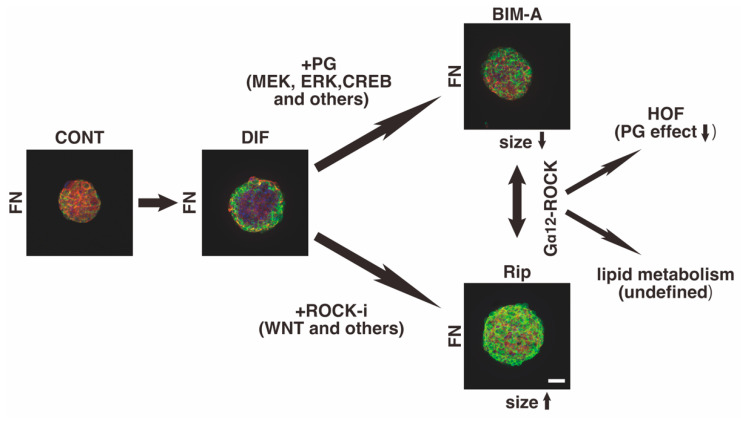
Hypothetical schema of PG and/or ROCK-i induced signaling toward 3D 3T3-L1 spheroids and experimental setups for the present study.

Within the field of ophthalmology, these PGs and ROCK-related signaling pathways have been recognized as therapeutic targets for the treatment of ocular diseases, especially glaucomatous optic neuropathy (GON) [17,18,19,20]. In fact, among the currently used glaucoma medications that function to decrease intraocular pressure (IOP), PGs are used as first-line drugs due to their powerful hypotensive effects with fewer systemic adverse effects [21]. After PGs, the second anti-glaucoma medication ROCK inhibitors (ROCK-i) and others are also available. However, recent reports have revealed that PGs induce local adverse effects on orbital fatty tissue, namely, a condition called “the deepening of the upper eyelid sulcus (DUES)”, and this is a concern for long-term users of PGs [22,23]. As possible mechanisms responsible for causing DUES, it has been suggested that orbital fat atrophy may be primarily involved in the development of this condition [24], although the precise molecular pathogenesis remains to be elucidated. Since orbital fatty tissue grows within a three-dimensional (3D) conical space, to establish a more suitable and representative in vitro model for the etiology of DUES, we employed a three-dimensional (3D) drop culture technique that replicates this type of adipocyte-spreading environment [25] using human orbital fibroblasts (HOFs) [26]. As a result, we found that our developed, in vitro, 3D HOFs spheroid model replicates DEUS pathogenesis, in that PGs induced a significant suppression of adipogenesis as well as causing the downsizing and stiffening of the 3D HOFs spheroids [27,28] (Figure 1). In contrast, compared with the PG-induced effects described above, ROCK-i induced opposite effects; that is, pan-ROCK-i, ripasudil hydrochloride hydrate (Rip), caused the enlargement and softening of the 3D HOFs spheroids [29] (Figure 1). In addition, in the case of the addition of ROCK-i (Rip) to PGs (bimatoprost acid, BIM-A), the effects that were induced by PGs on 3D HOFs spheroids were significantly cancelled [26] (Figure 1). Therefore, based upon these observations, we conclude that Rip may have some potential for serving as a second-line anti-glaucoma medication in addition to PGs, in avoiding the development of DUES that is induced by PGs. In addition, these observations also prompted us to facilitate additional scientific investigations related to the additive effects of ROCK-i to PGs within the area of lipid metabolism as well as metabolic syndrome, because both types of signaling are closely involved in the regulation of these mechanisms (Figure 1). In fact, in vitro, 3D spheroid cultured models have been extensively used in investigations designed to understand the etiology of metabolic syndromes such as obesity and type 2 diabetes [30]. In addition, our recently reported RNA sequence analysis of 3T3-L1 cells, which are the most frequently used preadipocytes in lipid research, demonstrated that there were significant differences between 2D planar cultures and 3D spheroid cultures [31]. In addition, our previous study revealed that ROCK-i induced similar effects on 3D 3T3-L1 spheroids—that is, enlargement and softening [32]—suggesting that ROCK-i may also have a substantial influence on lipid metabolism and may be related to the pathophysiology of metabolic syndrome. These findings stimulated us to further study ROCK-i induced effects, especially on cellular metabolic states, as well as its synergistic effects on other modulators, such as PGs.

Therefore, in the current study, to examine this issue further, we evaluated the effects of ROCK-i (Rip and/or Y27632) on a PG (BIM-A) on cellular metabolic states during adipogenesis, using a Seahorse Bioanalyzer on both 2D and 3D cultured 3T3-L1 preadipocytes, which are the most extensively characterized type of cells in the field of lipid research field. Additionally, several analyses including lipid staining, qPCR for adipocyte-related factors and the physical properties of the 3D spheroids were conducted.

As shown in these immunolabeled 3D 3T3-L1 spheroids with fibronectin (FN), the main component of the ECMs of the 3D spheroid, their sizes were increased upon adipogenic differentiation (DIF) as compared with those without DIF (CONT). Such DIF-induced effects were inhibited or facilitated by the PG, bimatoprost acid (BIM-A), or the ROCK inhibitor (ROCK-i), ripasudil (Rip), respectively. Although in the 3D spheroids of human orbital fibroblasts (HOFs), Rip suppressed these BIM-A-induced effects, such combined effects by PGs and ROCK-I on lipid metabolisms have not been elucidated yet. Scale bar: 100 μm. MEK: mitogen-activated protein kinase, ERK: extracellular signal-regulated kinase, CREB: the cyclic AMP response element binding protein.

## 2. Materials and Methods

### 2.1. Adipocyte Culture and Adipogenic Differentiation of 3T3-L1 Cells with or without PG and ROCK Inhibitors (ROCK-i)

Two-dimensional (2D) planar and three-dimensional (3D) spheroid cultures of 3T3-L1 preadipocytes (#EC86052701-G0, KAC, Osaka, Japan) and the induction of adipogenic differentiation were performed during 7 days, as described previously [32,33]. To study the drug efficacy of PG and ROCK-i, optimum concentrations of bimatoprost acid (BIM-A, 100 nM) with or without ROCK-is, Ripasudil (Rip, 10 µM) or Y27632 (10 µM), confirmed in our previous studies [32,33], were supplemented.

### 2.2. Lipid Staining by Oil Red O (2D) or BODIPY (3D)

Lipid staining of the 2D cultured 3T3-L1 cells by an Oil Red O staining assay was performed using a commercial kit (#133102; Abcam, Cambridge, UK). Microscopy images were obtained with a Nikon A1 confocal microscope (Tokyo, Japan) and their quantification was performed by measuring the optical density (O.D.) of the dissolved dye at 500 nm.

Alternatively, the lipid staining of the 4% paraformaldehyde (PFA)-fixed 3D spheroids was processed in a mixture of 0.1% BODIPY (#D3922; Thermo Fisher Scientific, Waltham, MA, USA), 0.1% DAPI (#D523; Doujin, Tokyo, Japan) and 0.1% phalloidin (#20553; Funakoshi, Tokyo, Japan) in phosphate-buffered saline (PBS) containing 3% bovine serum albumin (BSA) for 3 h. Their fluorescence intensity was measured using a Nikon A1 confocal microscope (Tokyo, Japan) and quantified using the Image J software version 2.0.0 (NIH, Bethesda, MD, USA).

### 2.3. Measurement of Real-Time Cellular Metabolic Functions

The oxygen consumption rate (OCR) and extracellular acidification rate (ECAR) of the 2D 3T3-L1 cells were measured using a Seahorse XFe96 Bioanalyzer (Agilent Technologies, Santa Clara, CA, USA), as described previously, with minor modifications [34,35]. Briefly, 20 × 10^3^ 2D 3T3-L1 cells were placed in the wells of a 96-well assay plate in the absence or presence of BIM-A and/or ROCK-i, as above. After replacing the culture medium with a Seahorse XF DMEM assay medium (pH 7.4, #103575-100; Agilent Technologies), supplemented with 5.5 mM glucose, 2.0 mM glutamine and 1.0 mM sodium pyruvate, basal OCR and ECAR values were determined using a Seahorse XFe96 Bioanalyzer and thereafter, the samples were further analyzed after supplementation with 2.0 μM oligomycin, 5.0 μM carbonyl cyanide p-trifluoromethoxyphenylhydrazone (FCCP), 1.0 μM rotenone and antimycin A, and 10 mM 2-deoxyglucose (2-DG). The OCR and ECAR values were normalized to the amount of protein per well.

### 2.4. Physical Properties, Size and Stiffness Analyses of the 3D 3T3-L1 Spheroids

The configuration of the 3D spheroids was observed by phase contrast (PC; Nikon ECLIPSE TS2, Tokyo, Japan) [27]. The resulting photograph of the spheroid was converted into an 8-bit image and the outer circumference of the spheroid was measured using the Image J software version 1.51n (National Institutes of Health, Bethesda, MD, USA), and the area within the circumference was determined as the size of the spheroid. Alternatively, the stiffness (μN/μm), as determined by the force (μN) required to compress the diameter of the 3T3-L1 spheroids by 50% (μm), was determined by a micro-indentation force analysis using a micro-squeezer (CellScale, Waterloo, ON, Canada), as described in a previous study [25].

### 2.5. Immunostaining of 3D 3T3-L1 Spheroids

The 3D 3T3-L1 spheroids were immune-stained using a primary antibody (1:200 dilutions), a rabbit anti-collagen monoclonal antibody (collagen 1; #600-401-103-0.1, collagen 4; #600-401-106-0.1, or collagen 6; #600-401-108-0.1; Rockland Immuno-Chemicals Inc., Pottstown, PA, USA) or a mouse anti-FN monoclonal antibody (#G0717; Santa Cruz Biotechnology, Santa Cruz, CA, USA), and a secondary antibody (1:500 dilutions) of goat anti-rabbit Alexa Fluor 488 IgG (#A-11070; Thermo Fischer Scientific, MA, USA) or goat anti-mouse Alexa Fluor 594 IgG (#A-11020; Thermo Fischer Scientific, MA, USA), with Alexa Fluor 594 phalloidin (#20553; Funakoshi, Tokyo, Japan) and DAPI (#D523; Dojindo, Kumamoto, Japan), at 1:1000 dilutions, for 3 h at room temperature, as described previously [33]. The fluorescence intensity of each ECM labeling procedure was determined using a Nikon A1 confocal microscope (Tokyo, Japan) and quantified using the Image J software version 2.0.0 (NIH, Bethesda, MD, USA).

### 2.6. Other Analytical Methods

Quantitative PCR was performed using predesigned specific primers and statistical analyses, using Graph Pad Prism 8 (GraphPad Software, San Diego, CA, USA), were performed as described previously [32,33]. For an analysis of the difference between groups, a grouped analysis with a two-way analysis of variance (ANOVA) followed by a Tukey’s multiple comparison test was performed. Data are presented as the arithmetic means ± the standard error of the mean (SEM).

## 3. Results

### 3.1. Additive Effects of Rock-Is on PGs on 2D Cultured 3T3-L1 Cells

In our preceding study, we found that the clinically used ROCK-i, Rip, significantly suppressed the effects of BIM-A on 3D human orbital fibroblasts (HOFs) spheroids. The fact that BIM-A induces the most prominent effect on human orbital fatty tissues [36,37] among the currently used PGs, suggests a possible crosslink between ROCK-i and PGs within adipocytes [26]. In the current study, to obtain currently unidentified insights into the crosslinking effect of ROCK-i and PGs toward fatty tissues in the case of the addition of ROCK-i to PG, we examined the additive effects of ROCK-i (Rip or Y27632) to BIM-A on the adipogenesis of 2D and 3D cultured 3T3-L1 cells, the most frequently used adipocyte in adipocyte-related research [38].

#### 3.1.1. Lipid Staining with Oil Red O and the Quantitative PCR of Adipogenesis (DIF+)-Related Genes, including *Pparγ*, Ap2 and Leptin (Figure 2)

Oil Red O lipid staining and the gene expression of the DIF+-related genes in the 2D cultured 3T3-L1 cells were significantly enhanced upon adipogenesis, and markedly suppressed by BIM-A, except for the expressions of *Ap2*, as described in our previous studies [32,33,39]. Upon the addition of ROCK-i, the staining intensities for Oil Red O again increased significantly. In contrast, however, the expressions of *Pparγ*, *Ap2* and *Leptin* were further markedly decreased or slightly increased by the addition of Rip or Y27632, respectively.

#### 3.1.2. The Quantitative PCR Analysis of ECMs including Collagen (Col) 1, 4 and 6, and Fibronectin (Fn) (Figure 2), and Real-Time Cellular Metabolic Analysis (Figure 3)

**Figure 3:**
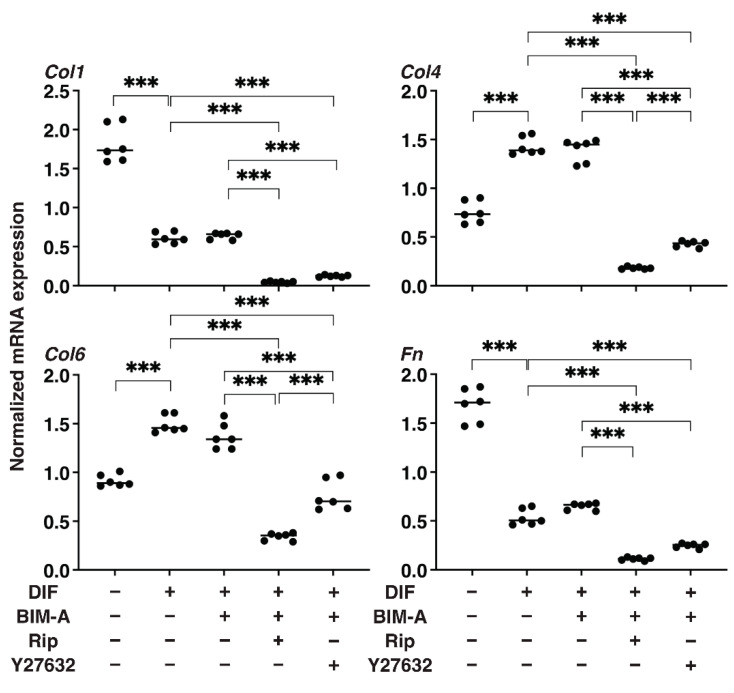
Additional effects of ROCK-i on BIM-A on the mRNA expression of ECMs of 2D planar cultured 3T3-L1 cells. The 2D planar cultures of 3T3-L1 cells were cultured under several conditions: preadipocytes of 3T3-L1 cells or their adipogenic differentiation (DIF) with or without the combination of 100 nM BIM-A (BIM-A) and ROCK-i (10 µM Ripasudil (Rip) or 10 µM Y27632). These specimens were subjected to qPCR analysis to estimate the mRNA expression of major ECMs (*Col*: collagen, *Fn*: fibronectin). All experiments were performed in triplicate using fresh preparations, each consisting of 5 samples. Data are presented as the arithmetic means ± the standard error of the mean (SEM). *** *p* < 0.005 (ANOVA followed by Tukey’s multiple comparison test).

**Figure 4:**
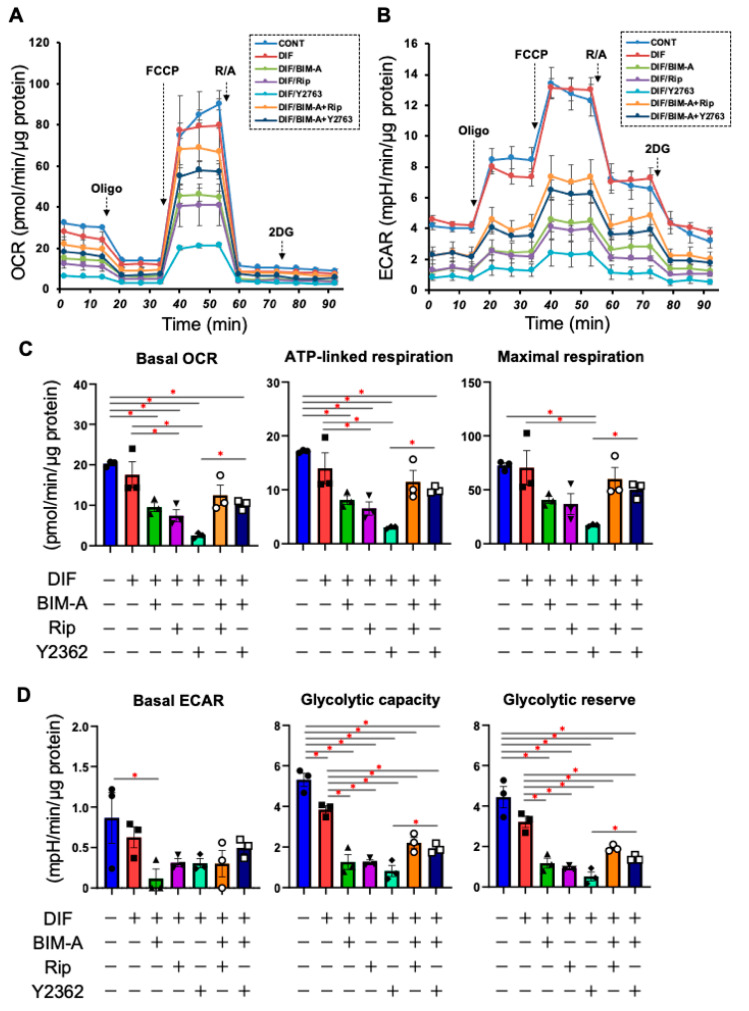
Additional effects of ROCK-i on BIM-A on the real-time cellular metabolic analysis of 2D planar cultured 3T3-L1 cells. The 3T3-L1 cells were 2D-cultured under several conditions: preadipocytes of 3T3-L1 cells (CONT) and their adipogenic differentiation (DIF) in the presence or absence of 100 nM BIM-A (BIM-A) and/or ROCK-i (10 µM Ripasudil (Rip) or 10 µM Y27632) were examined via real-time metabolic function analysis using a Seahorse XFe96 Bioanalyzer. The oxygen consumption rate (OCR, panel (**A**)) and extracellular acidification rate (ECAR, panel (**B**)) were measured, and thereafter, they were further measured after subsequent supplementation with oligomycin (a complex V inhibitor), FCCP (a protonophore), and rotenone/antimycin A (complex I/III inhibitors) and 2DG (a hexokinase inhibitor). The main parameters of the cellular metabolic analysis are shown in panels (**C**,**D**), respectively. Basal OCR was calculated by subtracting the OCR with rotenone/antimycin A from the OCR at baseline. ATP-linked respiration was analyzed by subtracting the OCR with oligomycin from the OCR at baseline. Maximal respiration was calculated by subtracting the OCR with rotenone/antimycin A from the OCR with FCCP. The basal ECAR was calculated by subtracting the ECAR with 2DG from the ECAR at baseline. Glycolytic capacity was calculated by subtracting the ECAR with 2DG from the ECAR with oligomycin. The glycolytic reserve was calculated by subtracting the ECAR at baseline from the ECAR with oligomycin. All experiments were performed in triplicate using fresh preparations (n = 3). Data are presented as the mean ± the standard error of the mean (SEM). * *p* < 0.05 (ANOVA followed by a Tukey’s multiple comparison test).

In terms of the mRNA expression of ECMs (Figure 3), a marked downregulation or upregulation was observed in *Col1* and *Fn*, or *Col4* and *Col6*, respectively, upon DIF+ addition. In the presence of BIM-A, although the DIF+-induced changes in these ECMs were not significantly altered, all four ECM molecules were substantially downregulated by the addition of ROCK-i to BIM-A. Real-time cellular metabolic measurements (Figure 4) demonstrated that although both OCR and ECAR were significantly reduced by a mono-treatment with either BIM-A and ROCK-i, the values were, to the contrary, enhanced in the case of BIM-A and Y27632. Therefore, these collective results suggest that some unknown synergy, and not simply the additive effects of ROCK-i and BIM-A, may be at play here.

### 3.2. Additive Effects of ROCK-Is to PGs on 2D Cultured 3T3-L1 Cells

Since the nature of adipogenesis of the 3T3-L1 cells was significantly different between the 2D planar cultures and 3D spheroid culture conditions [31], we also studied the additive effect of ROCK-i to BIM-A on DIF+ in 3D 3T3-L1 spheroids that were prepared as describe in a previous study [27,33].

#### 3.2.1. The 3D 3T3-L1 Spheroid Maturation and Their Physical Properties, Size (Figure 4) and Stiffness (Figure 5)

**Figure 5:**
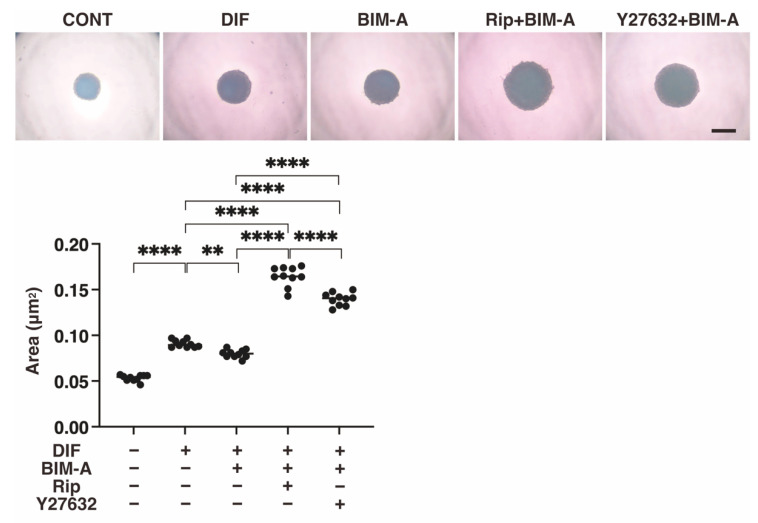
Additional effects of ROCK-i on BIM-A on the mean area sizes of the 3T3-L1 3D spheroids. The 3D spheroids of 3T3-L1 cells were cultured under several conditions: preadipocytes of 3T3-L1 cells (CONT) or their adipogenic differentiation (DIF) with or without the combination of 100 nM BIM-A (BIM-A) and ROCK-i (10 µM Ripasudil (Rip) or 10 µM Y27632). The mean area sizes (μm^2^) of the spheroids were measured and compared among experimental groups on Day 7. All experiments were performed in triplicate using fresh preparations, each consisting of 16 spheroids. Data are presented as the arithmetic means ± the standard error of the mean (SEM). ** *p* < 0.01, **** *p* < 0.001 (ANOVA followed by a Tukey’s multiple comparison test).

**Figure 6:**
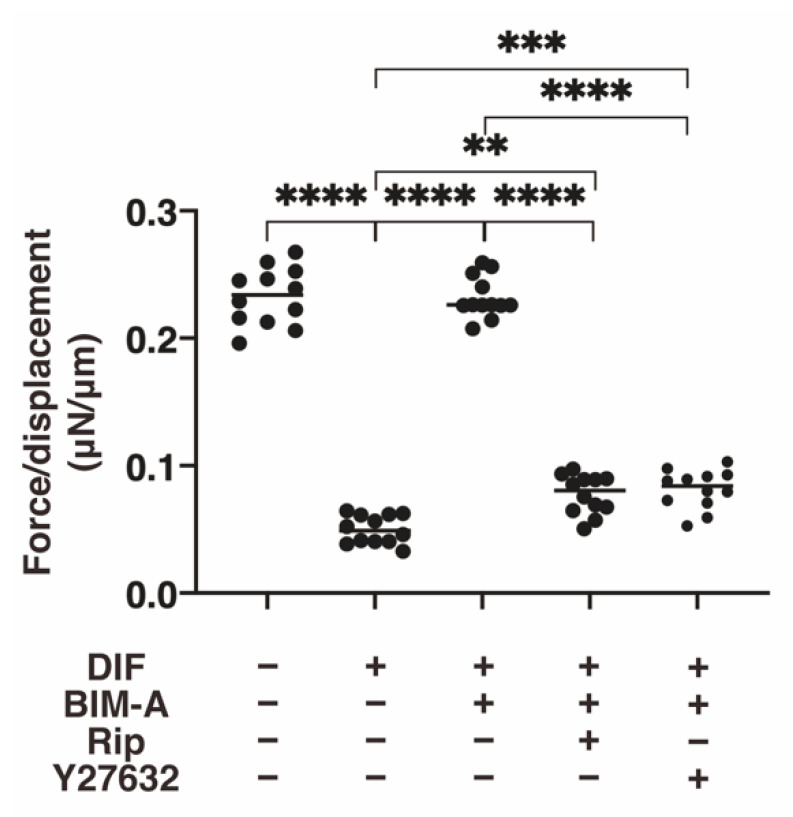
Additional effects of ROCK-i on BIM-A on physical stiffness during the adipogenesis of the 3T3-L1 3D spheroids. The 3D spheroids of 3T3-L1 cells were cultured under several conditions: preadipocytes of 3T3-L1 cells or their adipogenic differentiation (DIF) in the presence or absence of the combination of 100 nM BIM-A (BIM-A) and ROCK-i (10 µM Ripasudil (Rip) or 10 µM Y27632). The specimens collected on Day 7 were subjected to a physical solidity analysis. Among the above experimental conditions, the requiring force (μN) was measured and force/displacement (μN/μm) was potted (right panel). ** *p* < 0.01, *** *p* < 0.005, **** *p* < 0.001 (ANOVA followed by a Tukey’s multiple comparison test).

The following results were consistent with the results reported in a previous study: (1) DIF-induced 3D 3T3-L1 spheroids became smaller during the 7-day culture; (2) upon adipogenesis (DIF+), their mean area sizes became significantly larger; and (3) such DIF+-induced effects were significantly inhibited in the presence of 100 nM BIM-A. The addition of 10 μM ROCK-i to 100 nM BIM-A induced a substantial increase in the sizes of 3D spheroids until Day 7 (Figure 5). Concerning the micro-squeezer analysis, the 3D 3T3-L1 spheroids became physically softened upon adipogenesis (DIF+), and this stiffness was substantially increased or decreased by the treatment of BIM-A or BIM-A/and ROCK-I (Figure 6).

#### 3.2.2. Lipid Staining with BODIPY and the Quantitative PCR of Adipogenesis (DIF+)-Related Genes, including *Pparγ*, Ap2 and Leptin (Figure 6)

**Figure 7:**
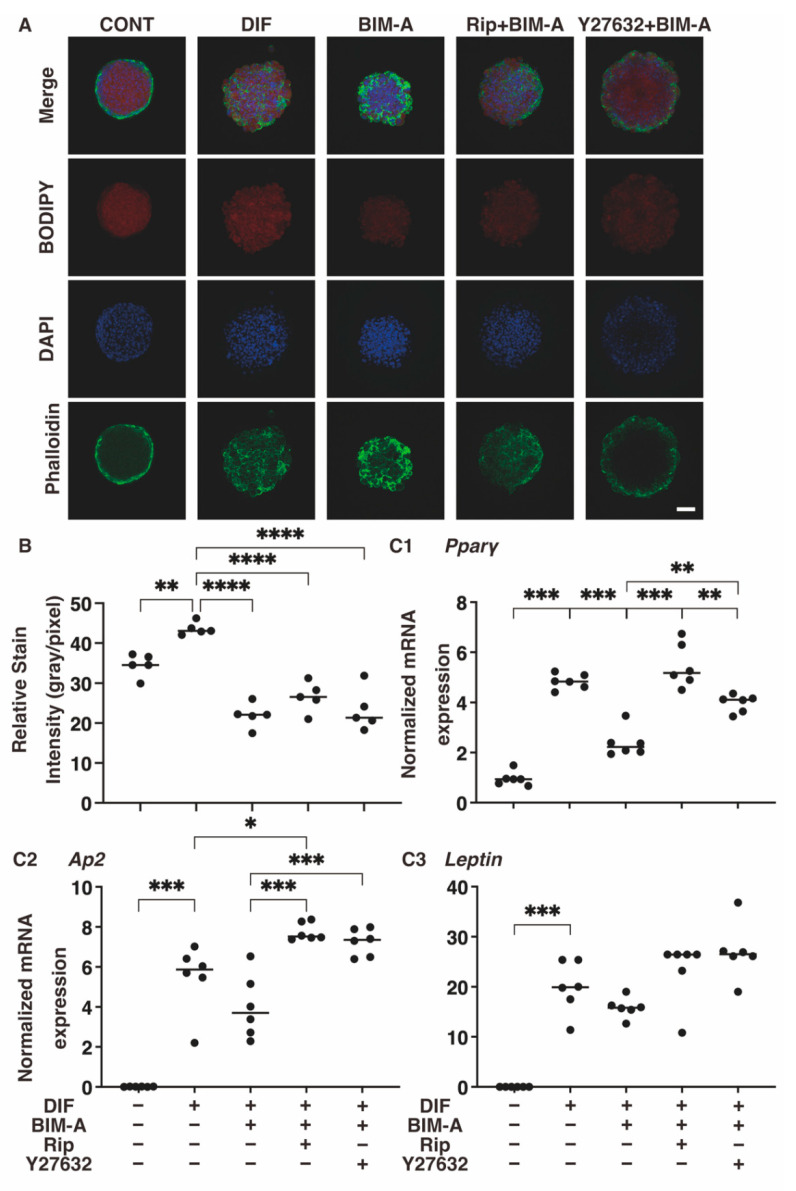
Additional effects of ROCK-i on BIM-A during the adipogenesis of 3T3-L1 3D spheroids. The 3D spheroids of 3T3-L1 cells were prepared under several conditions: preadipocytes of 3T3-L1 cells (CONT) or their adipogenic differentiation (DIF) with or without the combination of 100 nM BIM-A (BIM-A) and ROCK-i (10 µM Ripasudil (Rip) or 10 µM Y27632). These were immunostained by DAPI (blue), phalloidin (green) and BODIPY (red). Merge and BODIPY images are shown in panel (**A**) (scale bar: 100 μm) and their staining intensities (gray/pixel) were plotted (panel (**B**)). The mRNA expressions of adipogenesis-related genes, including *Pparγ* and *Leptin*, under the above conditions were plotted in panel (**C1**–**C3**). All experiments were performed in duplicate using fresh preparations, each consisting of 16 spheroids. Data are presented as the arithmetic means ± the standard error of the mean (SEM). * *p* < 0.05, ** *p* < 0.01, *** *p* < 0.005, **** *p* < 0.001 (ANOVA followed by a Tukey’s multiple comparison test).

As shown in Figure 7A,B, the DIF+-induced enhancement of the BODIPY staining intensities was significantly inhibited in the presence of BIM-A, while no change was observed upon the addition of ROCK-i to BIM-A. The mRNA expression of the adipogenesis-related genes was also significantly increased with DIF+. However, inconsistent with the BODIPY staining results, the addition of ROCK-i to BIM-A caused an enhancement in the mRNA expression of these adipogenesis-related genes (Figure 7(C1–C3)).

#### 3.2.3. The Expression of ECMs including Collagen (Col) 1, 4 and 6, and Fibronectin (Fn) Analyzed by qPCR (Figure 8) and Immunocytochemistry (Figure 9)

**Figure 8:**
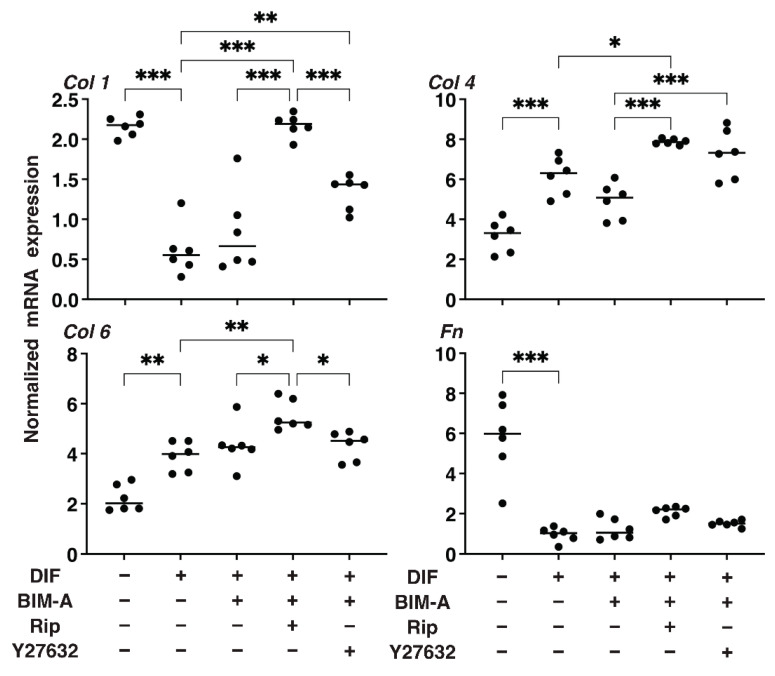
Additional effects of ROCK-i on BIM-A on the ECM-related mRNA expression of 3T3-L1 3D spheroids. The 3D spheroids of 3T3-L1 cells were cultured under several conditions: preadipocytes of 3T3-L1 cells or their adipogenic differentiation (DIF) in the presence or absence of the combination of 100 nM BIM-A (BIM-A) and ROCK-i (10 µM Ripasudil (Rip) or 10 µM Y27632). The specimens collected on Day 7 were subjected to a qPCR analysis to estimate the mRNA expression of ECMs (*Col*: collagen, *Fn*: fibronectin). All experiments were performed in duplicate using fresh preparations, each of which consisted of 16 spheroids. Data are presented as the arithmetic means ± the standard error of the mean (SEM). * *p* < 0.05, ** *p* < 0.01, *** *p* < 0.005 (ANOVA followed by a Tukey’s multiple comparison test).

Concerning the mRNA expression of ECMs (Figure 8), BIM-A caused significant changes in the DIF+-induced downregulation of *Col1* and *Fn,* and the upregulation of *Col4* and *Col6*, similar to the results of the 2D cell culture experiment described above. However, the additive effects were also different between the 2D and 3D spheroids. That is, the addition of Rip to BIM-A caused a significant increase in *Col1*, *Col4*, and *Col6* and a relative increase in *Fn*, but Y27632 caused a significant increase only in Col4. In terms of this discrepancy for the ECM expressions between the 2D and 3D cell cultures, we previously reported that the nature of 2D and 3D cultures was significantly different based upon their trypsin sensitivity [33] as well as the efficacy of adipogenesis [32].

**Figure 9:**
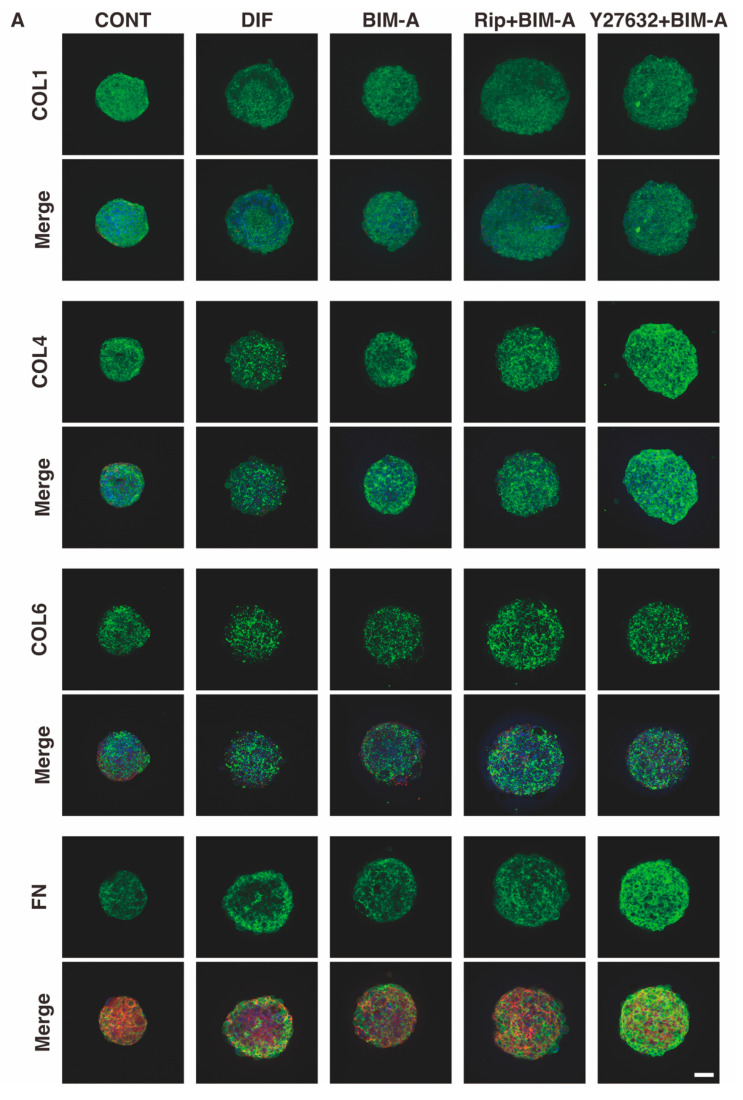

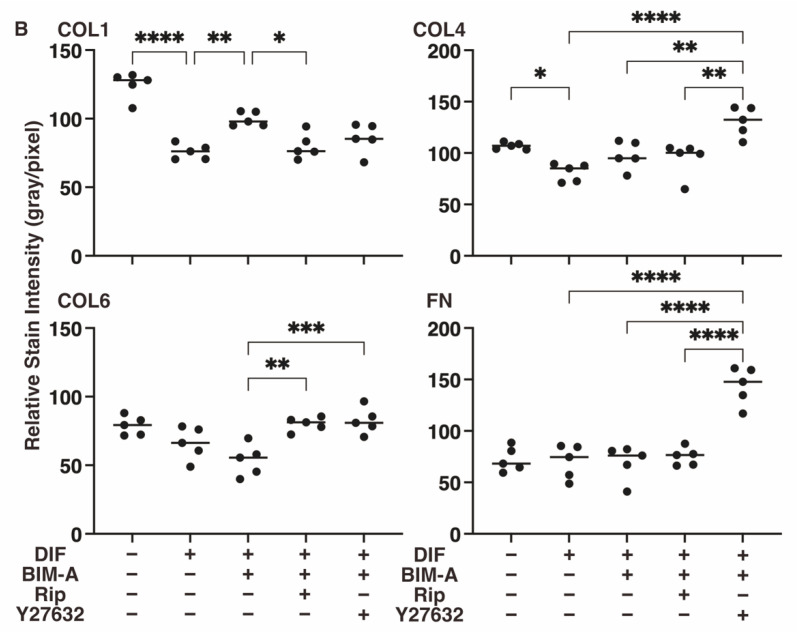
Representative confocal images showing the expression of ECMs in 3D 3T3-L1 spheroids under several sets of conditions. (**A**) On Day 7, the 3D cultures of spheroids of 3T3-L1 preadipocytes as the control (CONT), and their adipogenic differentiation in the absence (DIF) or presence of 100 nM bimatoprost free acid (BIM-A) and/or 10 μM ROCK-i (Rip or Y27632), were immunostained with specific antibodies of ECMs designated by the green color. Scale bar: 100 μm. (**B**) The staining intensities of the ECMs of the spheroids that were stained as above are plotted. All experiments were performed in duplicate using fresh preparations consisting of 5 spheroids each. Data are presented as the arithmetic mean ± the standard error of the mean (SEM). * *p* < 0.05, ** *p* < 0.01, *** *p* < 0.005, **** *p* < 0.001 (ANOVA followed by a Tukey’s multiple comparison test).

In contrast to the mRNA expression data, immunolabeling indicated that: (1) the levels of COL1 or COL4 significantly decreased upon the DIF+ treatment; (2) the addition of BIM-A to DIF+ induced a significant increase in COL1; (3) the addition of Rip to BIM-A caused a decrease in COL1 and an increase in COL6 levels; and (4) the addition of Y27632 to BIM-A induced an increase in all ECMs, except for COL1 (Figure 9). These discrepancies between gene expression and immunostaining can be attributed to the spatial distribution of the individual ECM molecules that are expressed within the 3D conformation of the spheroid, as was suggested in a previous study [33].

These collective results indicate that although the BIM-A-treated 3D spheroids were small and densely packed with lipids, the addition of ROCK-i to BIM-A induced 3D 3T3-L1 spheroids with greatly increased sizes, presumably due to the rich Cols framework as compared to 3D spheroids without ROCK-I and comparable amounts of lipids within both 3D spheroids.

## 4. Discussion

Since orbital fatty tissue grows within a three-dimensional (3D) conic space, our group recently established a more suitable and representative in vitro model using a three-dimensional (3D) drop culture technique to replicate this adipocyte-spreading environment [25], using human orbital fibroblasts (HOFs) [27,28] as well as 3T3-L1 cells [33], which are the most extensively characterized cells that are used in adipogenesis-related research [40]. Härmä et al. recently reported that the use of automated image analysis for the evaluation of phenotypic or morphometric aspects facilitated cell-based 3D assays in basic research as well as in drug discovery and target validation [41]. In an independent study, we also demonstrated that the characterization of the physical properties, sizes and stiffness of the 3D spheroids could also be used for the evaluation of several drug-induced effects [26,27,28,32,33]. For example, most recently, we found that ROCK-i significantly inhibited the downsizing and hardening effects of 3D HOFs spheroids that were induced by a PG, namely, bimatoprost acid (BIM-A) [26]. However, and quite interestingly, we also found that the biological features of the 3D spheroids were different between HOFs and 3T3-L1 cells; that is, DIF+ induced an increase or decrease in the stiffness of HOFs [26,27,28] and 3T3-L1 cells [32,33], respectively. These facts rationally suggest that the additive effects of ROCK-i on PGs may also be different between HOFs and 3T3-L1 cells.

Adipogenic differentiation from precursor stem cells to adipocytes is thought to be accomplished by two steps: determination and terminal differentiation [42]. Among these, the terminal differentiation step is relatively well identified as compared to the determination step. It has also been revealed that such adipogenesis processes are critically regulated by several key genes; that is, essential regulators of adipogenesis, the peroxisome proliferator-activated receptor γ (*PPARγ*) and CCAAT enhancer-binding protein α (C/EBPα) induce the expression of metabolic genes, including glucose transporter 4 (GLUT4), fatty acid binding protein 4 (FABP4) and leptin [42]. Throughout these gene functions, lipid droplets begin to appear within the cytoplasm and grow into mature adipocytes [42]. This adipogenesis process is known to also be modulated by several extracellular signaling proteins, including insulin/IGF-1, TGFβ, WNT10b, FGF, BMPs, PGs and others. Among these, PG-induced suppression of adipogenesis has begun to draw a lot of attention as a possible DUES etiology in the ophthalmology field. In addition, a significant contribution of ROCK signaling toward lipid adipogenesis has also been suggested [16]. That is, it was shown that the Rho/ROCK signaling pathway negatively regulates adipocyte differentiation, in which several underlying mechanisms, including a mechanism mediated by WNT genes, the regulation of actinomyosin formation, which is a key determinant of adipogenesis, the inhibition of the insulin requirement, and others have been suggested. However, such studies regarding ROCK signaling as it relates to adipogenesis have not been accomplished. Furthermore, except for our previous study using HOFs [26], no additional studies have been reported suggesting the existence of a possible crosslink between ROCK and PG signaling within lipid adipogenesis. In the current study, using a universal adipocyte cell line, 3T3-L1 cells, we confirmed that a crosslink exists in both signaling within lipid adipogenesis in general based upon the observations that the addition of ROCK-i cancelled the BIM-induced suppression of the several DIF+ structural and functional properties in both 2D and 3D cultured 3T3-L1 cells.

In terms of the anti-adipogenic effect of PGF2α, the FP-receptor-activated mitogen-activated protein kinase/extracellular signal-regulated kinase, the (MEK)/extracellular signal-regulated kinase (ERK) cascade and the binding of the cyclic AMP response element (CRE) binding protein (CREB) to the COX-2 promoter are known to be involved. Interestingly, in addition to this pathway, it is also known that the activation of the FP receptor by PGF2α is linked to the Gα12-ROCK signaling pathway. This observation suggests that the underlying mechanism may cause a crosslinkage between ROCK-i and PGs on adipogenesis that was reported in the current study. To support this possibility, Gα12 signaling appears to be involved in the regulation of the growth of skeletal muscle by modulating the metabolism of ECMs, similar to the current observation that pan-ROCK-is, Rip or Y27632 caused significant changes in the physical properties of the 3T3-L1 spheroids by modulating the expression of ECM molecules in addition to adipogenesis [32] (Table 1). Interestingly, in the case in which combinations of BIM-A and ROCK-i were used, in addition to some of the changes being able to be rationally explained by their independent effects, other currently unexplained and unidentified changes (designated as N.I.) were also recognized (Table 1). More interestingly, such combined effects by BIM-A and ROCK-i were also different between the 3T3-L1 cells and HOFs [26] (Table 1). Therefore, given the above collective findings, these findings strongly support the existence of relationships between ROCK-i and PGs on adipogenesis. Thus, the mechanisms responsible for such unidentified relationships will need to be investigated in a future project.

**Figure 10:**
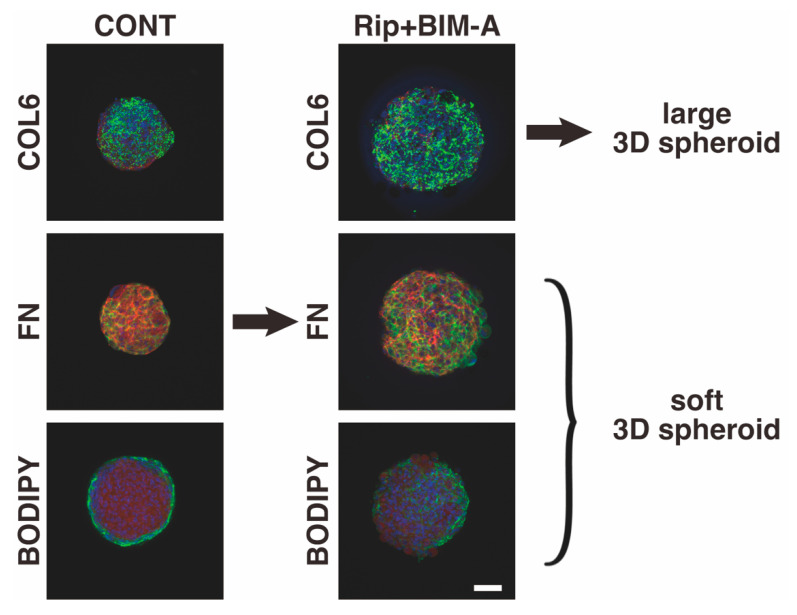
Hypothetical model of the mechanism responsible for inducing huge-sized and soft 3D spheroids caused by the addition of Rip to BIM-A.

In conclusion, in the current study, we report on new observations to suggest that the addition of ROCK-i cancelled the PG-induced suppression of adipogenesis in 3T3-L1 cells, resulting in the formation of greatly enlarged 3D spheroids. This points to additional insights into our understanding of lipid-related research in general. However, the current study also had the following limitations: (1) It is known that there are two functionally diverse isoforms of ROCKs, ROCK 1 and ROCK 2, in a variety of tissues, including adipocytes [43,44,45,46,47,48]. Among these, ROCK2, but not ROCK1, is thought to be responsible for the anti-adipogenic activity of Rho in 3T3-L1 and mouse embryonic fibroblasts (MEFs) [49,50], in which ROCK1 and/or ROCK2 are responsible for the unidentified crosslinking with PGs. In fact, the ROCK2-i, KD025, induced quite different effects with PGs in 3D HOFs spheroids [26]. However, as shown above, since the drug-induced effects by ROCK-is and/or BIM-A also are substantially different between 3T3-L1 and HOFs (Table 1), additional studies using siRNA or isotype-specific inhibitors for ROCK 1 or 2, to determine which isoform among ROCK1 and ROCK2 plays a key role, will need to be carried out. (2) The mechanism responsible for causing such large and soft 3D spheroids upon the addition of ROCK-i to BIM-A has not been identified yet. As a possible mechanism, we speculate that the additive effects of ROCK-i to BIM-A induced: (1) a significant upregulation of COL6, which mainly makes up the structural frame of the 3D spheroid, and (2) relatively insufficient amounts of lipid and FN content within the 3D spheroid may contribute to the formation of such large-sized and soft 3D spheroids, as shown in Figure 10. In support of this speculation, it has been reported that ROCK contributes to the actin cytoskeleton and fibronectin matrix assembly [51,52,53], and that ROCK-i alters cytoskeletal arrangement and cell shape [54,55]. In addition, we also found that ROCK-i only caused the formation of large and soft 3D 3T3-L1 spheroids as was observed in our previous study [32], the effects of which were similar, but less intense, compared to the combination of ROCK-i and PG [32]. However, real-time cellular metabolic measurements by a Seahorse Bioanalyzer identified some quite new and interesting results, suggesting the existence of some unknown type of synergy, and that not simply the additive effects of ROCK-i and BIM-A are involved in this process. Therefore, to elucidate the underlying mechanisms responsible for this, we also plan to perform RNA sequence analyses to identify the types of upstream and downstream regulations that are involved in this process.

As shown in the immunolabeled 3D spheroids, a significant upregulation of collagen6 (COL6) as the main component of the structural frame of the 3D spheroid, and relatively insufficient amounts of lipid and fibronectin (FN) content within the 3D spheroid, represent a possible mechanism for the formation of the huge-sized and soft 3D spheroids upon the administration of ROCK-i to BIM-A. Scale bar: 100 μm. CONT: 3T3-L1 preadipocyte, Rip+BIM-A: adipogenic differentiated 3T3-L1 in the presence of R-p and BIM-A, BODIPY: lipid staining by BODIPY.

## Figures and Tables

**Figure 2:**
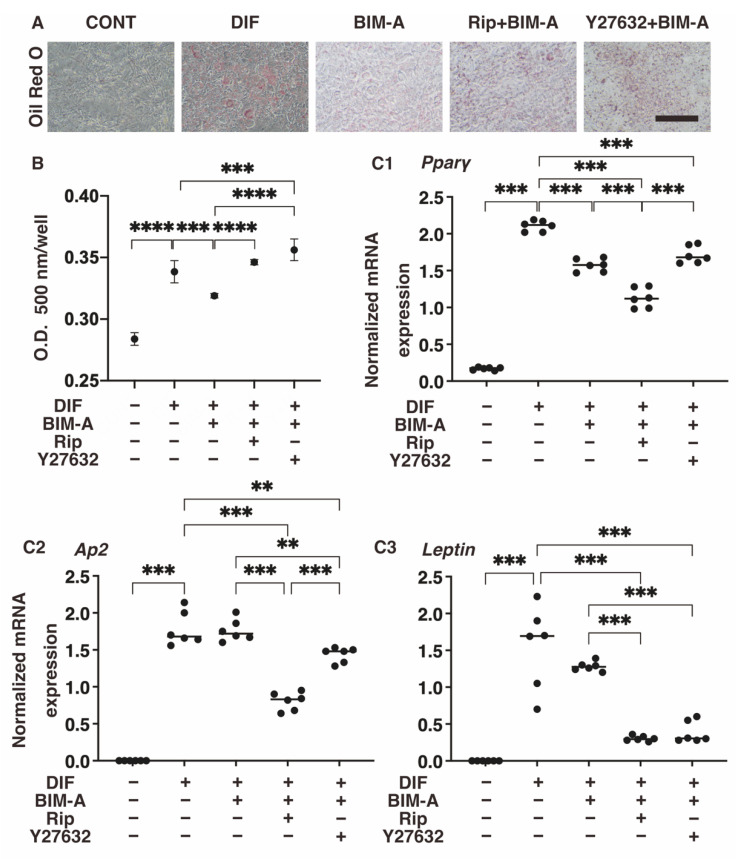
Additional effects of ROCK-i on BIM-A during the adipogenesis of 2D planar cultured 3T3-L1 cells. The 2D planar cultures of 3T3-L1 cells were cultured under several conditions: preadipocytes of 3T3-L1 cells (CONT) or their adipogenic differentiation (DIF) with or without the combination of 100 nM BIM-A (BIM-A) and ROCK-i (10 µM Ripasudil (Rip) or 10 µM Y27632). These specimens were subjected to analysis by Oil Red O lipid staining (panel (**A**); representative phase contrast images, scale bar: 100 μm, and panel (**B**); their staining intensities, O.D.) and qPCR of the master adipogenesis gene, *Pparγ* (panel (**C1**–**C3**)). All experiments were performed in triplicate using fresh preparations, each consisting of 5 specimens. Data are presented as the arithmetic means ± the standard error of the mean (SEM). ** *p* < 0.01, *** *p* < 0.005, **** *p* < 0.001 (ANOVA followed by a Tukey’s multiple comparison test).

**Table 1:** Summary of the effects of mono- or combined treatments of PGs and ROCK inhibitors on the physical properties of the 3D 3T3-L1 spheroids, and adipogenesis and gene expression in the 2D planar and 3D spheroid cultured 3T3-L1 cells and human orbital fibroblasts (HOFs).

		BIM-A		Rip		Y27632	BIM-A+Rip				BIM-A+Y27632	
		L1	HOF **	L1 *	HOF **	L1 *	L1		HOF **		L1	
size 3D		↓	↓↓	↑	→	↑	↑	(B < R)	↓↓	(B > R)	↑	(B < Y)
stiffness 3D		↑	↑↑	↓	↓	↓	↓	(B < R)	↓	(B < R)	↓	(B < Y)
lipid stain	2D	↓		↑		↑	→	(B = R)			↑	(B < Y)
	3D	↓	↓↓	→	↑	↑	↓	(B > R)	→	(B = R)	↓	(B > Y)
*Pparγ*	2D	↓		→		↑	↓↓	N. I.			↓	(B > Y)
	3D	↓↓↓	↓	→	→	↑↑	→	N. I.	→	N.I.	↓↓	(B > Y)
*Ap2*	2D	→		→		→	↓↓↓	N. I.			↓	N. I.
	3D	→	↓	↑↑	↑↑	↑↑↑↑	↑	N. I.	↑↑	(B < R)	→	N. I.
*Leptin*	2D	→		↓↓↓		→	↓↓↓	(B < R)			↓↓↓	N. I.
	3D	→		↑		↑↑↑	→	N. I.			→	N. I.
*Col1*	2D	→		↓		→	↓	(B < R)			↓	N. I.
	3D	→	↑↑	↑	→	→	→	N. I.	↑↑	(B > R)	→	N. I.
*Col4*	2D	→		↓		→	↓	(B < R)			↓	N. I.
	3D	→	→	↑	↑↑	↑	→	N. I.	→	N. I.	↓	N. I.
*Col6*	2D	→		→		↑	↓	N. I.			↓	N. I.
	3D	→	→	↑	→	↑	↑	(B < R)	↑	N. I.	→	N. I.
*Fn*	2D	→		→		→	↓	N. I.			↓	N. I.
	3D	→	→	→	→	→	↑	N. I.	→	B=R	↑	N. I.

BIM-A: bimatoprost acid, Rip: ripasudil, 2D: two-dimensional planar culture, 3D: three-dimensional spheroid culture, *Pparγ*: peroxisome proliferator-activated receptor γ, *Ap2*: adipocyte protein 2, *Col*: collagen, *Fn*: fibronectin, →: no significant change, ↑: significant increase (*p* < 0.05), ↑↑: significant increase (*p* < 0.01), ↑↑↑: significant increase (*p* < 0.005), ↓: significant decrease (*p* < 0.05), ↓↓: significant decrease (*p* < 0.01), ↓↓↓: significant decrease (*p* < 0.005), ↑↑↑↑: significant decrease (*p* < 0.001), * results for Rip and Y27632 from 3T3-L1 cells [17] and ** HOF-related data [10] were taken from our previous studies. Parentheses indicate the effects of combinations of BIM-A (B), and Rip (R) or Y27632 (Y), and can be explained by their independent actions; for example, B < R refers to the finding that the effect of B was higher than for R, or cannot be explained because these were affected non-independently together (N. I.).

## Data Availability

Not applicable.
